# Vaccination scenario-based study on seasonal influenza in Republic of Korea

**DOI:** 10.1371/journal.pone.0322686

**Published:** 2026-04-20

**Authors:** Jongmin Lee, Vijay Pal Bajiya, Eunok Jung

**Affiliations:** Department of Mathematics, Konkuk University, Seoul, Korea; Soongsil University, KOREA, REPUBLIC OF

## Abstract

**Background:**

Seasonal influenza presents a persistent threat to global health, with the elderly (65 + years) facing disproportionate risks of severe clinical outcomes. While the World Health Organization advocates for targeted immunization, the epidemiological impact of varying vaccination schedules remains a critical area for quantitative evaluation. This study investigates how the prioritization and temporal shifting of vaccine administration influence disease burden within the demographic context of Korea.

**Method:**

We constructed an age-structured compartment model across four demographic groups (G1:0–14, G2:15–49, G3:50–64, and G4: 65+) using 2023–2024 seasonal influenza data in Korea. Among the various scenarios evaluated, we specifically performed an analysis by advancing the vaccination start date for the elderly group (G4) by up to two weeks to evaluate its comparative effectiveness against the baseline Korean National Immunization Program (KNIP). Furthermore, to quantify parameter uncertainty and assess the robustness of our model, we performed bootstrapping and partial rank correlation coefficient (PRCC) analyses.

**Results:**

Our analysis reveals that, under the baseline vaccination strategy, the highest infection rates occur in the G1 age group, while early vaccination of the G4 group is found to be effective in reducing hospitalizations and deaths. Specifically, scenarios prioritizing early vaccination for the elderly (G4) with different immune states were associated with a 34.2% reduction in cumulative cases (range: 11.5–53.9%) and a 39.1% decrease in peak infection levels (range: 12.3–60.4%) compared to the baseline. Sensitivity analysis using PRCC showed that the symptomatic infectious period and G4 transmission parameters were identified as the primary drivers of cases and deaths.

**Conclusion:**

These findings suggest that targeted, timely vaccination of the elderly (65 + years) can contribute to mitigating the overall epidemic burden and alleviating periods of high hospital demand. Our simulation results indicate that age-specific strategies, particularly those accelerating vaccination for the elderly, offer a valuable quantitative framework for reducing the public health impact of seasonal influenza. While these outcomes are subject to model-specific assumptions, they provide a reasonable basis for further refining immunization programs within structured epidemic interventions.

## Introduction

Influenza remains a major public health challenge across the globe, with a particularly significant impact on older adults aged 65 years and older. Every year, the virus affects between 5% and 10% of the adult population and 20% to 30% of children worldwide [1]. It is responsible for causing between 3 and 5 million cases of severe illness, along with an estimated 290,000–650,000 deaths annually [2]. In the Republic of Korea, influenza leads to around 400,000 outpatient visits and approximately 7,000 hospitalizations each year [3,4]. Older adults in the country face a disproportionately high risk, with a mortality rate of 46.98 per 100,000, a figure that is far greater than the general population’s mortality rate of 5.97 per 100,000 [3]. This stark difference underscores the elevated vulnerability of elderly individuals to the complications of influenza, highlighting the urgent need for targeted prevention and treatment strategies for this demographic.

The influenza vaccination program in Korea, as part of the KNIP, primarily targets specific high-risk groups to prevent the seasonal flu. These groups include children under the age of 13, adults aged 65 and older, and pregnant women, who are all considered particularly vulnerable to the flu [5]. The program aims to ensure these high-risk individuals receive the vaccine to minimize the impact of the flu season. In terms of high-risk populations for seasonal influenza, the focus is on children younger than 5 years old, adults aged 50 years and older, pregnant women, and individuals suffering from chronic health conditions [5]. These groups are more likely to experience severe complications from the flu, and vaccinating them is a priority to protect public health. The proportion of the general public that has been inoculated against influenza during the 2023–2024 season is currently estimated at 40% overall, according to aggregated national data on vaccination coverage. However, coverage rates are higher in certain groups: around 82% of adults aged 65 and older have received the vaccine, and approximately 70% of children under 13 have been vaccinated [6]. The government’s strategy includes starting the vaccination process earlier in the season, with vaccinations for children under 13 typically beginning in the first week of October and those for adults aged 65 and older beginning in the third week of October [6]. This approach aims to ensure early protection for the most vulnerable populations before the peak of the flu season.

Given the potential pandemic scenarios and challenges in vaccine production [7], early data on age-specific incidence and mortality can help prioritize vaccine distribution and optimize disease burden reduction. Chowell et al. [8] investigated an age-structured model, where the transmission rate for each age group was assumed to be influenced by contact rates. The age-specific risk of illness and hospitalization rates were estimated based on incidence data. Their findings indicate that an adaptive strategy targeting individuals aged 6–59 years is the most effective approach for reducing hospitalizations and deaths. Matteo Ratti et al. [9] investigated an SEIR epidemic model to assess optimal vaccination strategies for seasonal influenza in a nursing home with a long-term care (LTC) environment. They incorporated contact matrices based on surveys of both healthcare workers (HCWs) and residents. Their findings indicate that prioritizing the vaccination of residents, rather than healthcare workers, may still be the most effective preventive approach. Diana and Gergely [10] explored an age-structured compartmental model with vaccination status to assess the impact of age-specific vaccination scheduling during a pandemic influenza outbreak. Their findings highlighted that the timing of vaccination by age group can significantly influence the epidemic’s outcome. Knipl and Roast [11] explored an age-structured compartmental model to assess the impact of age-specific vaccination during a pandemic, finding that targeted scheduling can significantly affect the epidemic outcome, with delays worsening the attack rate. Mylius et al. [12] developed a model for the Asian flu, recommending prioritizing high-risk individuals when a vaccine becomes available, and targeting high-transmission groups if vaccination starts early. Mantel et al. [13] evaluated seasonal flu vaccination in Belarus, Morocco, and Thailand, emphasizing health workers as a key target group due to their role in influencing acceptance and ensuring vaccine delivery. McMorrow et al. [14] analyzed influenza data from South Africa to prioritize risk groups for vaccination. Tsuzuki et al. [15] found that vaccinating children under 15 would have a greater epidemiological impact than targeting the elderly, though Japan’s current program focuses on older individuals. Other studies have emphasized the importance of age-specific intervention strategies and have examined the distribution of vaccines across different age groups, assuming pre-seasonal vaccination [12,16,17]. Kim et al. primarily focused on the 2009 A/H1N1 pandemic period to suggest optimal pharmaceutical and nonpharmaceutical interventions using optimal control theory [18] or to determine region specific vaccination priorities [19]. Song et al. demonstrated through mathematical modeling that switching from standard to adjuvanted influenza vaccines for the elderly becomes cost effective when efficacy surpasses a threshold of 56.1% [20]. Distinct from these pandemic centered analyses, our research focuses on non-pandemic periods to address the epidemiological interplay between vaccination timing and prioritization while accounting for actual healthcare system capacity. Our model incorporates a KNIP-specific age structure and accounts for the waning of vaccine-induced immunity, thereby capturing the potential for breakthrough infections. Crucially, we differentiate our work by integrating realistic hospital bed capacities as a core simulation outcome, rather than focusing solely on disease transmission. By providing data grounded in actual medical resource constraints, our study offers practical insights directly relevant to healthcare professionals and policymakers. Based on our modeling, we explore vaccination strategies to propose effective alternatives to the current approach in Korea, aiming to reduce influenza cases and evaluate the impact of targeted efforts across diverse demographic segments.

## Materials and methods

### Data source

We utilized data on influenza cases from August 2023 to March 2024, sourced from the Health Insurance Review and Assessment (HIRA) [21,22] and WHO Flunet [23], to develop an age-structured model aimed at examining the transmission dynamics of seasonal influenza in Korea. We used vaccination data from the annual report of the KDCA [6,24], which provides weekly vaccination numbers. According to the KNIP for the 2023–2024 season, vaccination coverage is higher in certain groups: approximately 82% of adults aged 65 and older, around 70% of children under 13, and 133,735 out of 265,262 eligible pregnant women have been vaccinated [6]. It is assumed that vaccination coverage remains consistent across various vaccination scenarios. We composed the contact matrix *C*_*jk*_ of four age groups from the POLYMOD study [25] considering the number of populations in each age group. The details on the contact matrix are attached in S1 File.

### Mathematical model

We categorized the total population into four distinct age groups aligned with the KNIP strategy: Group 1 (G1, 1–14 years), Group 2 (G2, 15–49 years, including pregnant women), Group 3 (G3, 50–64 years), and Group 4 (G4, 65 years and older). The population is divided into compartments based on health and vaccination status, where the subscript *j* denotes the specific age group (*j* = 1, 2, 3, 4). The compartments are defined as follows: Susceptible (*S*_*j*_), Exposed (*E*_*j*_), Infected (comprising asymptomatic *A*_*j*_, symptomatic *I*_*j*_, and hospitalized *H*_*j*_), Recovered (*R*_*j*_), and Deceased (*D*_*j*_). Vaccination status is tracked through three compartments: ineffectively vaccinated (*U*_*j*_), effectively vaccinated (*V*_*j*_), and fully protected (*P*_*j*_). The total population for the *j*^th^ age group is given by $N_{j}=S_{j}+U_{j}+V_{j}+P_{j}+E_{j}+A_{j}+I_{j}+H_{j}+R_{j}$, such that $N=\sum_{j=1}^{4}N_{j}$.


$$\begin{array}{cc} \frac{dS_{j}}{dt}= & \;\;-u_{j}(t)-\beta (t)\sum_{k=1}^{4}C_{jk}\frac{I_{k}+\delta A_{k}}{N}S_{j}+\gamma P_{j}\\ \frac{dE_{j}}{dt}= & \;\;\beta (t)\sum_{k=1}^{4}C_{jk}\frac{I_{k}+\delta A_{k}}{N}(S_{j}+U_{j}+V_{j})-\kappa E_{j}\\ \frac{dI_{j}}{dt}= & \;\;q\kappa E_{j}-(1-q_{hj})\alpha I_{j}-q_{hj}\rho I_{j}\\ \frac{dH_{j}}{dt}= & \;\;q_{hj}\rho I_{j}-(\sigma_{j}+d_{j})H_{j}\\ \frac{dA_{j}}{dt}= & \;\;(1-q)\kappa E_{j}-\eta A_{j}\\ \frac{dR_{j}}{dt}= & \;\;\eta A_{j}+(1-q_{hj})\alpha I_{j}+\sigma_{j}H_{j}\\ \frac{dD_{j}}{dt}= & \;\;d_{j}H_{j}\\ \frac{dU_{j}}{dt}= & \;\;(1-\epsilon_{j})u_{j}(t)-\beta (t)\sum_{k=1}^{4}C_{jk}\frac{I_{k}+\delta A_{k}}{N}U_{j}\\ \frac{dV_{j}}{dt}= & \;\;\epsilon_{j}u_{j}(t)-\beta (t)\sum_{k=1}^{4}C_{jk}\frac{I_{k}+\delta A_{k}}{N}V_{j}-\omega V_{j}\\ \frac{dP_{j}}{dt}= & \;\;\omega V_{j}-\gamma P_{j} \end{array}$$ (1)

**Fig 1:**
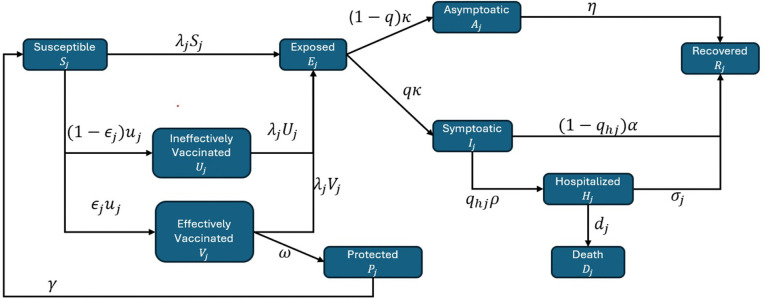
Flow diagram shows the transition of population across different compartments.

In the force of infection term $\begin{array}{c} \lambda_{j}(t)=\beta (t)\sum_{k=1}^{4}C_{jk}\frac{I_{k}\delta A_{k}}{N}\; \end{array}$ (Fig 1), *C*_*jk*_ represents the contact rate at which individuals in age group *j* come into contact with individuals in age group *k*, δ shows the relative infectiousness of asymptomatic compared to symptomatic individuals, and $\begin{array}{c} \beta (t)=\beta_{j}{\left(1\alpha_{0}\cos\left(\frac{t-t_{0}}{52}\right)\right)}^{1s} \end{array}$ represents the seasonal transmission rate. $\beta_{j}$ represents the transmission coefficient for *j*^th^ age group. *t*_0_ and $\alpha_{0}$ indicate the phase and amplitude of seasonality, respectively. *s* represents the sharpness of seasonality in the transmission of influenza, controlling *t*he curvature of the seasonal transition curve.

The average duration of the latent period and delay before hospitalization are assumed to be represented by 1/κ and 1/ρ, respectively. The proportion of infected individuals who exhibit symptoms of influenza is denoted by *q*. Additionally, it is assumed that asymptomatic infected individuals recover at a rate η without requiring hospital care. Furthermore, only *q*_*hj*_, the age-specific proportion of symptomatic infected individuals, requires hospitalization due to severe symptoms, while the remaining proportion recovers at a rate α without any hospital care. This model also incorporates age-specific recovery rates for hospitalized individuals $\sigma_{j}$ and mortality rates *d*_*j*_. Currently, according to the KNIP, the authority provides seasonal influenza vaccination for specific high-risk groups, including children under 13 years of age, adults over 65 years, and pregnant women. As a result, an age-specific vaccination rate *u*_*j*_ is considered. However, it is assumed that only $\epsilon_{j}$, the age-specific proportion of vaccinated individuals, receives an effective vaccine. The average duration to achieve immunity from the effective vaccine is 1/ω. It is also assumed that after 1/γ time duration, the protected individuals also lose immunity and again become susceptible to influenza. The following are key assumptions:

- We utilized a constant contact matrix derived from POLYMOD projections to represent age-specific interactions.
- Mixing within each of the four defined age groups is assumed to be homogeneous.
- Immunity acquired through natural infection is assumed to remain robust, with no significant waning within a single influenza season.

Note that our model explicitly accounts for the waning of vaccine-induced immunity. We assumed an average duration of vaccine protection of 180 days. Consequently, our model captures the potential for breakthrough infections, estimating that approximately 15.4% of effectively vaccinated individuals lose immunity and return to susceptibility within one month of vaccination


$${\left(1-\frac{1}{180}\right)}^{30}=84.6\%$$ (2)

### Epidemiological inputs

Vaccine efficacy varies by age, with lower effectiveness observed in the older population [26]. Based on the literature [20,27], we use the following vaccine effectiveness rates: 62.5% for G1, 54% for G2 and G3, and 47.8% for G4. Our model is calibrated using case data from the HIRA database in Korea, which also includes the age distribution of influenza cases. We estimate the transmission coefficient for different age groups $\beta_{j}$, the phase of seasonality *t*_0_, the sharpness of seasonality *s*, and the seasonali*t*y constant $\alpha_{0}$. The remaining model parameters are derived from published literatures, the same as given in Table 1. To quantify parameter uncertainty and assess the robustness of our model, we performed bootstrapping and partial rank correlation coefficient (PRCC) analyses, the results of which are detailed in S1 File.

**Table 1:** Numerical values and descriptions of model parameter.

Parameters	Description	Values				Ref.
$\frac{1}{\kappa }$	Duration of latent period (week)	$\frac{1.9}{7}$				[28,29]
*q*	Proportion of infected showing symptoms	0.67				[30]
η	Recovery rate of asymptomatic	1.167				[19]
	individuals (week^−1^)					
α	Recovery rate of symptomatic	1.167				[19]
	individuals (week^−1^)					
$\frac{1}{\omega }$	Duration for developing immunity (week)	$\frac{10}{7}$				
δ	Infectivity reduction factor	0.5				[28]
	for asymptomatic					
$\frac{1}{\gamma }$	Duration to losing the vaccination	$\frac{180}{7}$				[31]
	immunity (week)					
$\frac{1}{\rho }$	Delay in getting hospitalization (week)	$\frac{1}{7}$				[32,20]
		G1	G2	G3	G4	
*q*_*h*_	Proportion of symptomatic infected	0.011	0.01	0.018	0.04	[3,20]
	to be hospitalized					
σ	Recovery rate of hospitalized	1.454	1.505	1.47	1.306	[20,33]
	individuals (week^−1^)					
*d*	Death rate in hospitalized	0.0002	0.0007	0.0012	0.0392	[20,33]
	individuals (week^−1^)					

Since it is hard to estimate each compartment’s values at the beginning of an epidemic, August 2023, which has low and unchanged confirmed cases, is chosen to decide the values. In the equation (1), no change of the compartment *X* means dX/dt≃0 at that period, and the confirmed cases are $C_{j}=q\kappa E_{j}$ for the *j*^th^ age group. The calculation is attached in S1 File.

### Algorithm for least square method for data fitting

The transmission related parameters, $\beta_{j}$, $\alpha_{0}$, *s*, and *t*_0_, were estimated by fitting the model output $q\kappa E_{j}$ for *j* = 1,2,3,4 *t*o the observed number of influenza cases (1). To minimize the error between the model output and the actual influenza cases, we used the built-in *ga* and *fminsearch* function in MATLAB.

Step 1. Obtain the confirmed cases of model output at time *t* for every age group are as a function of parameters $\Theta =\{\beta_{1},\;\beta_{2},\;\beta_{3},\;\beta_{4},\;\alpha_{0},\;s,\;t_{0}\}$. It is given by

$$C_{j}(t,\Theta )=q\kappa E_{j},\;\;\;\;\text{where}\;\Theta =\{\beta_{1},\;\beta_{2},\;\beta_{3},\;\beta_{4},\;\alpha_{0},\;s,\;t_{0}\}\;\;\;\text{and}\;\;j=1,2,3,4.$$

For the initial values of the population of each age group, the calculation is attached in S1 File.Step 2. Compute the sum of squares of errors at each time point, given by

$$SS_{k}(\Theta )=\sum_{j=1}^{4}\sum_{i=1}^{m}{\left(C_{j}(t_{i})-C_{j}(t_{i},\Theta )\right)}^{2},$$

where, *C*_*j*_(*t*_*i*_) is the actual data at $t_{i}^{th}$ day and *m* is the number of data points. *k* represents the number of iterations performed such that *SS*_*k*_ is the sum of squares of errors in the *k*^th^ iteration.Step 3. Compute the value of parameter Θ such that

SS=min{SS(Θ)}.

### Vaccination scenario with different timing

**Fig 2:**
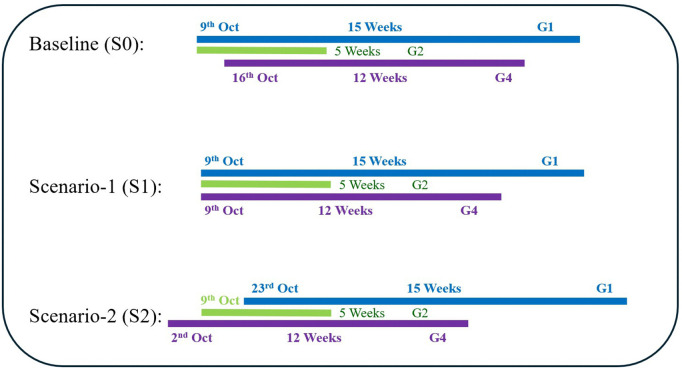
Three different vaccination scenarios based on the timing of vaccine in each group.

Three distinct vaccination scenarios are considered, each based on different timing strategies for administering the vaccine across various age groups. However, for all scenarios, the duration of vaccination and the total number of vaccines administered remain constant for each age group. The current vaccination strategy, as outlined by the KNIP in Korea, is used as the baseline vaccination scenario (S0). In this baseline scenario, vaccination for the G1 group begins on October 9th, and for the G4 group, it starts on October 16th. In Vaccination Scenario-1 (S1), the timing of vaccination for each group follows the baseline strategy, with vaccinations starting on October 9th for all groups, as per the KNIP guidelines. In Vaccination Scenario-2 (S2), the G4 group is prioritized, and vaccination for this group begins 2 weeks earlier than in the baseline scenario, intending to assess the impact of an earlier vaccination start for this particular group. The different vaccination scenarios and specific timing for each group’s vaccination are clearly illustrated in a schematic diagram presented in Fig 2. This diagram provides a visual representation of the variations in vaccination timing across the three scenarios, offering a comprehensive overview of the strategies under consideration.

## Results

### Parameters estimation

**Fig 3:**
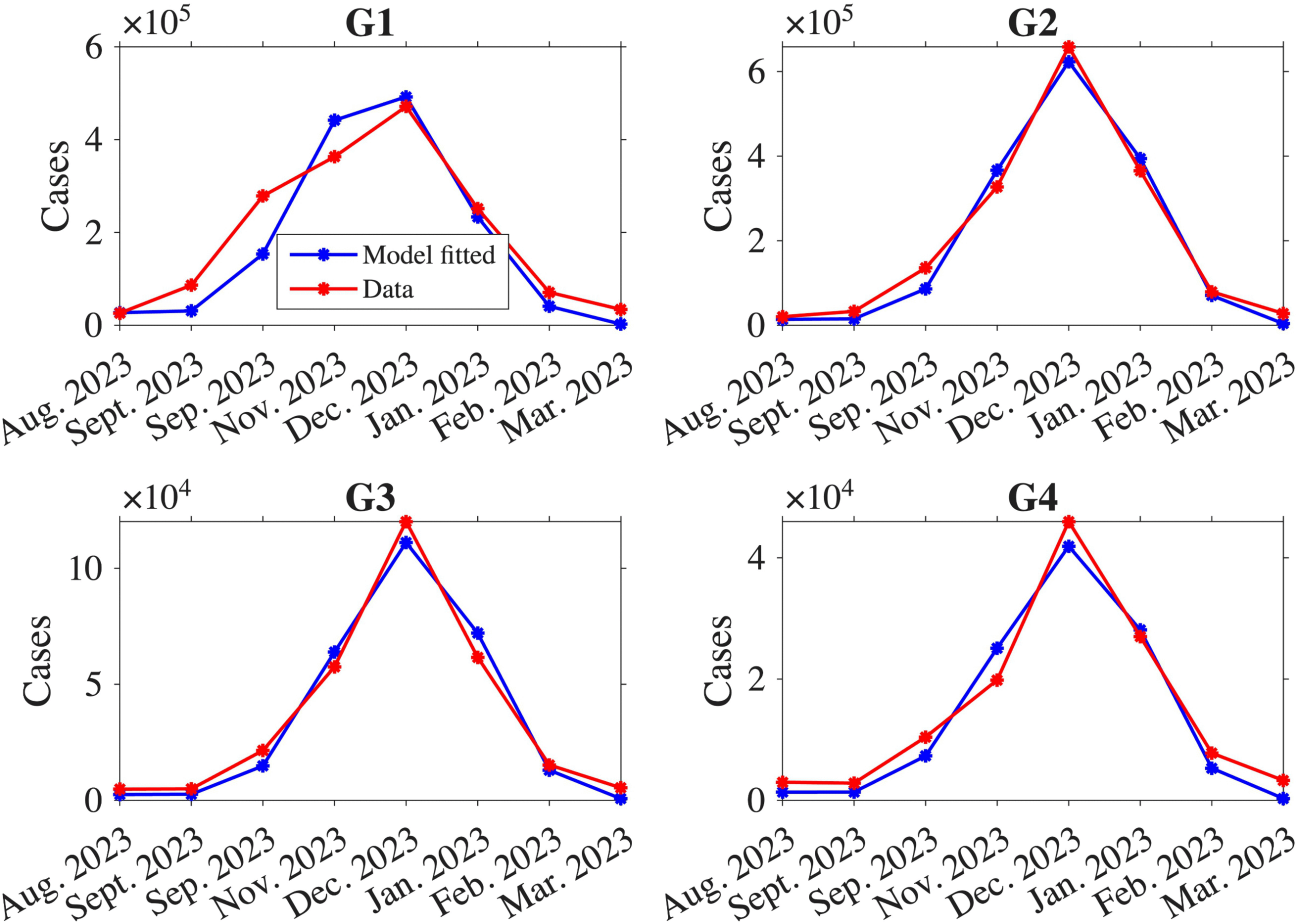
The model output is compared to actual data, with the number of influenza cases shown as red curves and the corresponding model output for the estimated parameters depicted as blue curves.

We obtain the value of the estimated parameters $\Theta =\{\beta_{1},\;\beta_{2},\;\beta_{3},\;\beta_{4},\;\alpha_{0},\;s,\;t_{0}\}$ for the best fitting of model output to actual data. The estimated parameters were obtained as follows: $\beta_{1}=3.7062,\;\beta_{2}=0.5346,\;\beta_{3}=0.1023,\;\beta_{4}=0.0203$, $\alpha_{0}=0.5743$, *s* = 0.9866, and *t*_0_ = 15.3738. The model fitting result is depicted in Fig 3. In our parameter estimation, we observed that the transmission coefficien*t* for G1 was the highest, followed by those for G2, G4, and G3. This pattern is consistent with the characteristic of influenza, which tends to spread more easily among younger individuals [34]. According to the HIRA data, 28.58% of individuals in G1 contracted the infection during a season, the highest rate among all age groups. In comparison, 7% of G2, 2.27% of G3, and 1.21% of G4 were infected during the simulation period in Korea. Our model also produces results that align with these trends (see Fig S1 in S1 File). Given the highest infection rate observed in G1, it follows that the transmission coefficient for this group is the highest, reflecting the increased susceptibility and transmission dynamics within this age group. The uncertainty analysis for the estimated parameters are included in (Fig S2) in S1 File.

### Impact of vaccination shifting

**Fig 4:**
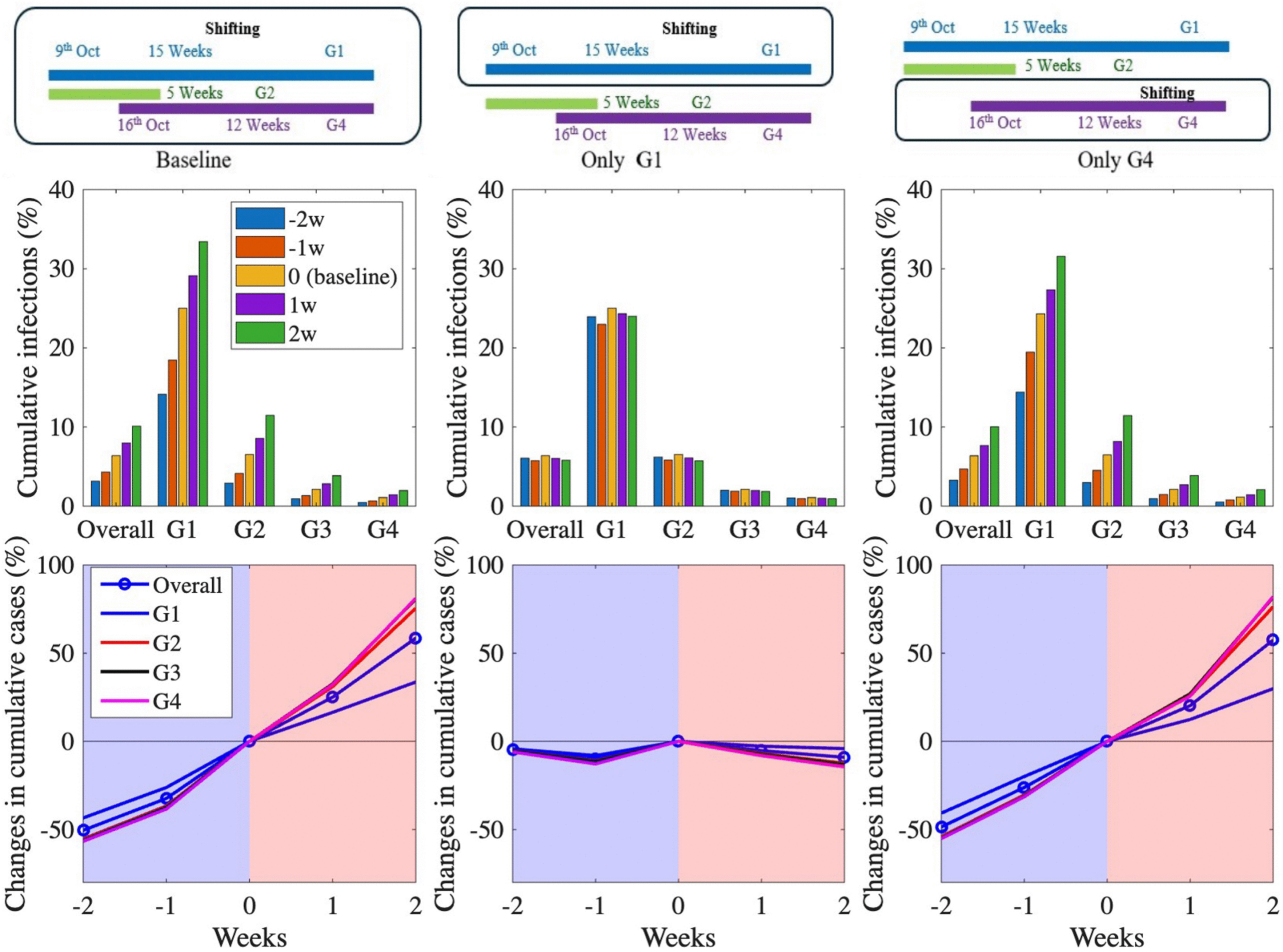
Impact of vaccination timing on cumulative infections (%) (top) and changes in cumulative cases (%) (bottom).

In Fig 4, we examined the impact of shifting vaccination timing on cumulative influenza cases by exploring three distinct scenarios: (1) shifting the baseline vaccination (i.e., the current vaccination strategy as per the KNIP), (2) shifting vaccination for the G1 group only, while keeping the vaccination strategy for other groups unchanged, and (3) shifting vaccination for the G4 group only, while maintaining the baseline vaccination strategy for the other groups. For each of these scenarios, we analyzed the effects of shifting vaccination by 2 weeks earlier, 1 week earlier, 1 week later, and 2 weeks later, and observed how these changes affected the cumulative cases across different age groups and the overall population.

When we shifted the baseline vaccination to 2 weeks earlier, we observed a substantial reduction in cumulative cases across all age groups, with approximately a 50% decrease for each group. However, when vaccination was delayed by 2 weeks, the increase in cumulative cases ranged from 33.7% to 81.1% across different age groups. The G4 group experienced the largest increase (81.1%), while the G1 group showed the smallest (33.7%). This relatively smaller increase in G1 may be attributed to the already high cumulative incidence in this group under the baseline strategy. In contrast, the G4 group maintains the lowest infection rate under the current strategy, making it more susceptible to a significant surge if vaccination is delayed.

When vaccination was shifted only to the G1 group, the changes in cumulative cases were relatively small. In contrast, when vaccination was shifted only for the G4 group, the observed changes in cumulative cases were similar to those seen when shifting the baseline vaccination strategy. Specifically, the G1 group showed less variation in cumulative cases when the vaccination shift was applied only to the G4 group, likely because the vaccination strategy for G1 remained unchanged, as per the baseline. Therefore, the relatively smaller impact on G1 cases can be attributed to the fact that no adjustment was made for this group’s vaccination timing. The similar impact of vaccination timing on the number of deaths across different groups can be observed in (Fig S3) in S1 File.

Our results underline the critical importance of vaccination timing for the G4 group in reducing Korea’s seasonal influenza burden. Specifically, prioritizing an earlier vaccination schedule for this demographic significantly decreases overall cases by leveraging their high vaccine uptake and low baseline infection rates. By effectively interrupting the chain of transmission, such a targeted approach offers a powerful strategy to manage seasonal outbreaks more efficiently than the current baseline.

### Vaccination scenarios based on timing

**Fig 5:**
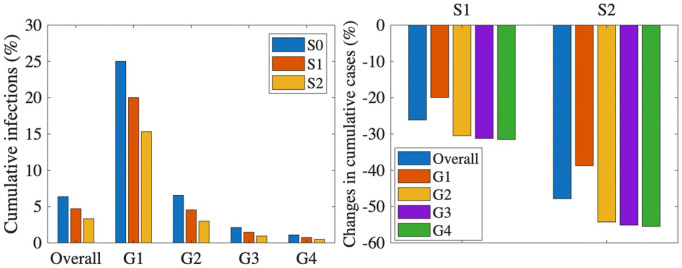
Cumulative cases for three different vaccine scenarios.

In the left panel of Fig 5, the percentage of cumulative cases for each age group, as well as the overall cumulative cases, is presented for each vaccination scenario. The right panel of Fig 5 shows the changes in cumulative cases for vaccination scenario 1 (S1) and scenario 2 (S2) in comparison to the baseline scenario.

Overall cumulative cases in the S1 scenario decreased by 26.1% compared to the baseline. In contrast, the S2 scenario demonstrated a more substantial reduction, with cumulative cases dropping by approximately 47.8%. This indicates that the S2 scenario results in a more significant reduction in cumulative cases across all age groups and the overall population than the baseline. Similar trends for cumulative deaths are observed in (Fig S4) in S1 File, where the S2 scenario exhibited a significant reduction of approximately 55%. The greater reduction observed in the S2 scenario suggests its effectiveness in curbing the spread of influenza. As a result, the S2 scenario offers a more effective strategy for reducing influenza cases in Korea compared to the baseline. By advancing the G4 group’s vaccination schedule by two weeks, this approach achieves a more substantial reduction in overall transmission. Therefore, implementing an earlier vaccination start for this demographic is expected to yield a considerable impact on mitigating the national influenza burden.

Table 2 presents a summary of the results of Fig 4 and 5, including the absolute values and the reduction in cumulative cases from the baseline scenario.

**Table 2:** Impact of varying vaccination timing on cumulative cases.

Scenario	Age group	2 weeks faster	1 week faster	Baseline	1 week later	2 weeks later
	Overall	1633067 (−50.5%)	2229107 (−32.4%)	3297666	4124883 (25.1%)	5228916 (58.6%)
	G1	782991 (−43.4%)	1021991 (−26.2%)	1384435	1611879 (16.4%)	1850416 (33.7%)
	G2	683680 (−55.4%)	969839 (−36.7%)	1531898	2007749 (31.1%)	2688750 (75.5%)
	G3	119646 (−56.2%)	170782 (−37.5%)	273349	362530 (32.6%)	494188 (80.8%)
All group	G4	46750 (−56.7%)	66495 (−38.4%)	107982	142725 (32.2%)	195561 (81.1%)
	Overall	3137421 (−4.9%)	2971677 (−9.9%)	3297666	3124642 (−5.2%)	2998728 (−9.1%)
	G1	1325414 (−4.3%)	1272811 (−8.1%)	1384435	1346318 (−2.8%)	1327654 (−4.1%)
	G2	1451946 (−5.2%)	1362567 (−11.1%)	1531898	1425383 (−7.0%)	1340566 (−12.5%)
	G3	258567 (−5.4%)	242144 (−11.4%)	273349	253688 (−7.2%)	238105 (−12.9%)
Only G1	G4	101494 (−6.0%)	94154 (−12.8%)	107982	99251 (−8.1%)	92401 (−14.4%)
	Overall	1695492 (−48.6%)	2433930 (−26.1%)	3295681	3963856 (20.3%)	5188798 (57.4%)
	G1	797748 (−40.7%)	1077301 (−20.0%)	1345863	1513026 (12.4%)	1748192 (29.9%)
	G2	699232 (−54.0%)	1057650 (−30.5%)	1520927	1913079 (25.8%)	2681147 (76.3%)
	G3	123217 (−54.9%)	187866 (−31.2%)	273178	347007 (27.0%)	496230 (81.7%)
Only G4	G4	50677 (−55.2%)	77547 (−31.5%)	113225	142342 (25.7%)	206145 (82.1%)

### Impact of vaccine effectiveness, coverage and timing in G4 group

**Fig 6:**
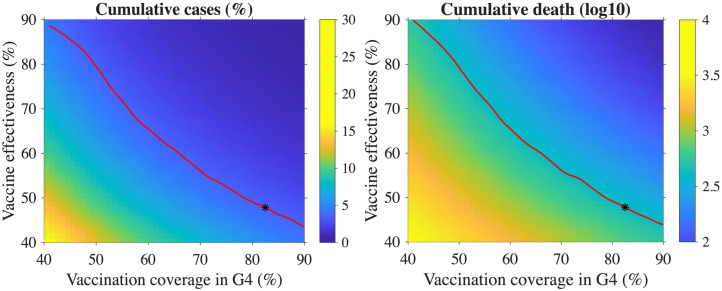
Cumulative cases percentage (left panel) and deaths in log10 scale (right panel) for different vaccine effectiveness and coverage in the G4 group are shown. The red curve represents the percentage of overall cumulative cases and cumulative deaths, which are identical to those observed in the S2 scenario. The black stars denote the default scenario.

In the Fig 4, it was observed that influenza cases are particularly more influenced by the vaccination of the G4 group. Fig 5 highlights that the S2 scenario is more effective in reducing influenza cases in Korea. Building on this, we further explore the impact of vaccine effectiveness, coverage, and timing specifically for the G4 group within the S2 scenario. In the S2 scenario, the vaccine coverage and effectiveness for the G4 group are assumed to be 82.5% and 47.8%, respectively. The left panel of Fig 6 shows that, even with vaccine coverage lower than that in the S2 scenario, influenza cases can still be reduced by increasing the vaccine effectiveness in the G4 group. This finding suggests that improving vaccine effectiveness may compensate for lower vaccine coverage. A similar pattern is observed in the right panel of Fig 6, where the reduction in influenza deaths follows a similar trend: increasing vaccine effectiveness leads to a decrease in the number of deaths, even with lower coverage. To increase vaccine effectiveness, alternative vaccines, such as the adjuvanted quadrivalent influenza vaccine, could be considered. This vaccine has higher efficacy than the standard quadrivalent influenza vaccine [27,35], and its use in the G4 group could further enhance the impact of vaccination efforts. By improving both the vaccine coverage and effectiveness, particularly with alternative vaccines, the reduction in influenza cases and deaths can be further optimized in the G4 group, thereby contributing to better control of the disease in Korea.

**Fig 7:**
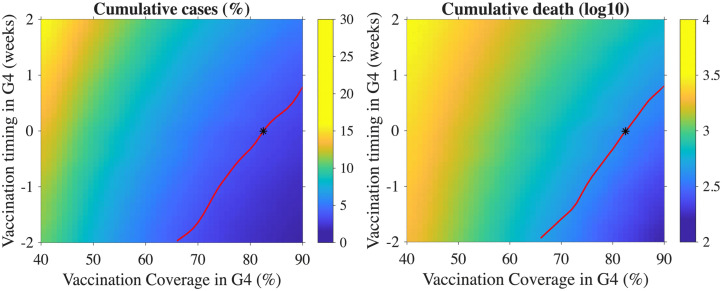
Cumulative cases percentage (left panel) and deaths in log10 scale (right panel) for different vaccine timing and coverage in the G4 group are shown. The red curve represents the percentage of overall cumulative cases and cumulative deaths, which are identical to those observed in the S2 scenario. The black stars denote the default scenario.

In the left panel of Fig 7, it can be observed that even with vaccine coverage lower than that in the S2 scenario, influenza cases can still be reduced by administering vaccination earlier in the G4 group. Specifically, by providing vaccination two weeks earlier than in the S2 scenario, the cumulative number of influenza cases can be reduced, even at a vaccination coverage of 67%. This demonstrates that advancing the vaccination timeline for the G4 group can lead to a significant reduction in the number of cases, even when the coverage is somewhat lower than in the S2 scenario.

A similar trend is evident in the right panel of Fig 7, where influenza deaths follow a comparable pattern. By shifting the vaccination timing for the G4 group to an earlier start, influenza-related deaths can also be reduced, mirroring the trend seen with cumulative cases. These results emphasize the importance of vaccination timing in reducing both influenza cases and deaths, particularly when earlier vaccination is implemented for the G4 group.

### Impact of natural immunity on peak

**Fig 8:**
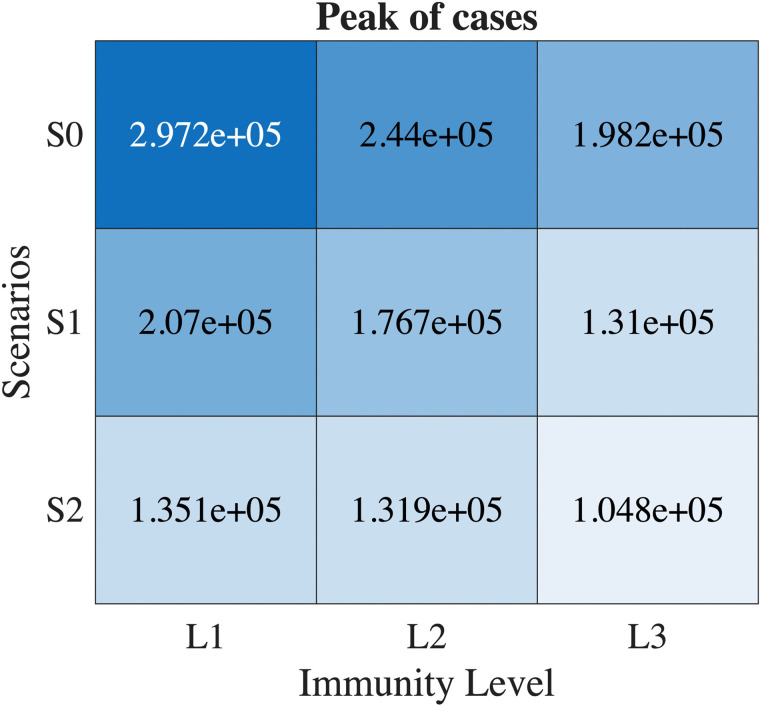
The peak size for different vaccination scenarios and immunity levels.

In this study, we consider three distinct levels of immunity: (1) L1: Immunity resulting from the reported cases of the previous year, (2) L2: Immunity resulting from both reported and unreported cases of the previous year, and (3) L3: Immunity from reported and unreported cases of the previous year, along with 50% of the cases from the year before that. Fig 8 illustrates the peak size of cumulative influenza cases under different vaccination scenarios and immunity levels. As expected, the model predicts the smallest peak in the S2 scenario when the L3 immunity level is applied. This suggests that a higher level of immunity leads to a substantial reduction in the peak of influenza cases in the S2 scenario. Table 3 presents the peak timing of cumulative cases for each vaccination scenario and immunity level. In the S0 scenario, the peak is delayed by one week as the immunity level increases from L1 to L2. In contrast, no changes in peak timing are observed for the S1 and S2 scenarios, regardless of the immunity level. This implies that the timing of the peak is relatively insensitive to variations in immunity levels under these specific vaccination strategies.

**Table 3:** Timing of peak for different vaccination scenarios and immunity levels (in which week the peak of cases attains).

Vaccination Scenarios				
Immunity		**S0**	**S1**	**S2**
	**L1**	19	20	20
	**L2**	20	20	20
	**L3**	20	20	20

**Fig 9:**
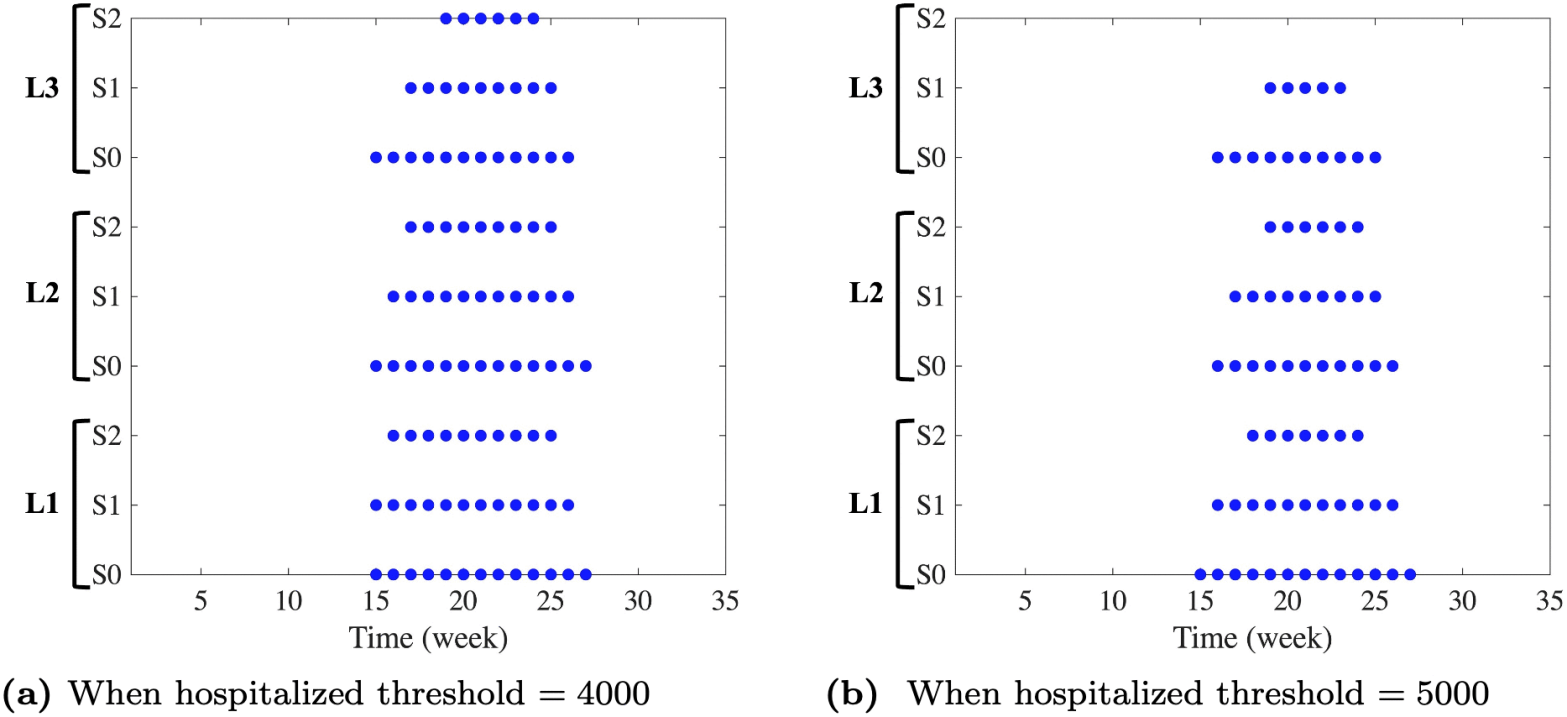
Time in which the hospitalized individuals exceed the particular threshold of those.

In our analysis, we examine two distinct thresholds for the number of hospitalized individuals: threshold-1 is set at 4000 and threshold-2 is set at 5000. These values were selected because they were chosen to lie between the number of isolation rooms (3032) and isolation wards (6687) available in Korea representing different levels of healthcare system strain. Note that since a single isolation room typically encompasses multiple isolation wards, the capacity thresholds were set at 4,000 and 5,000 to reflect this hierarchical structure. Fig 9 presents the duration over which the number of hospitalized individuals surpasses each of these thresholds. This figure allows us to observe how the burden on hospitals varies under different vaccination scenarios and immunity levels. As shown in Fig 9 and (Table S2) in S1 File, the duration for which hospitalized individuals exceed the thresholds is longest in the baseline scenario (S0). In contrast, scenario S2 consistently results in the shortest duration of hospital burden across all immunity levels considered. This suggests that S2 is the most effective in reducing influenza-related healthcare strain among the scenarios. Consequently, this strategy offers the most significant potential to mitigate the hospital burden, demonstrating its efficacy in controlling the impact of seasonal influenza on the healthcare system.

## Discussion

The potential for a devastating influenza pandemic, characterized by high rates of morbidity and mortality, particularly among the elderly, presents a significant challenge to global public health systems. While non-pharmaceutical interventions provide crucial time for response, policies to mitigate seasonal influenza must prioritize effective pharmacological measures. In this context, the efficient prioritization of vaccine distribution is a critical component, requiring careful consideration of population demographics and available medical resources

In this study, we developed a mathematical model to assess the transmission dynamics of seasonal influenza, taking into account age-related differences in disease susceptibility, transmission rates, hospitalization, and mortality. Our model incorporates time-dependent vaccination rates, allowing us to explore different vaccination strategies under varying conditions of immunity and vaccination timing. The 2017 POLYMOD contact matrix was selected over recently published Korean contact data [25] because the available Korean studies were conducted during or immediately after the COVID-19 pandemic [36,37] and therefore reflect altered contact behaviors rather than baseline, non-pandemic contact patterns.

Our results highlight that the highest infection rates were observed in the G1 age group (children aged 0–14 years), which is consistent with the increased susceptibility and heightened transmission dynamics within this age cohort. Under the baseline vaccination strategy, which aligns with the current KNIP in Korea, 25.16% of individuals in the G1 group, 7.90% in the G2 group (15–49 years), 2.34% in the G3 group (50–64 years), and 1.23% in the G4 group (65 years and older) were infected during the 2023–2024 flu season. These findings are consistent with data from the HIRA [21,22].

Our analysis further reveals that prioritizing vaccination for the G4 group (those aged 65+) can substantially reduce the burden of the epidemic. In scenarios where vaccination is prioritized for this age group (Scenario S2), cumulative cases decreased by approximately 47.8% and 26.1% reduction (S1), where no specific prioritization is implemented, compared to the baseline scenario. Advancing the vaccination schedule for the G4 group leads to a marked reduction in the peak number of cases, as individuals in this high-risk group contribute significantly to the severity of the epidemic. Even in scenarios with lower vaccine coverage than that of S2, improving the effectiveness of the vaccine for the G4 group can still lead to a notable reduction in influenza cases. This suggests that enhancing vaccine efficacy, for example, by using adjuvanted or alternative quadrivalent vaccines, could partially compensate for lower vaccine coverage in this critical age group [20,27].

The study demonstrates that the S2 strategy, which prioritizes vaccination for the G4 age group, has significant benefits for the community. This vaccination strategy reduces the peak number of influenza cases by 17.8 − 64.6% for different levels of immunity and reduces the period for which hospitals become overwhelmed. These findings highlight how early, targeted vaccination of high-risk populations is crucial for lessening the public health impact of seasonal influenza. These results offer actionable insights for developing age specific, optimal vaccination strategies that align with WHO guidelines to minimize public health impact. By focusing on the strategic distribution of vaccines, policymakers can develop more effective responses to seasonal outbreaks, ensuring that high priority populations are protected even within the constraints of limited hospital bed capacity.

Overall, this study contributes valuable insights into the design of age-specific vaccination strategies that reduce influenza incidence, hospitalizations, and mortality. By improving our understanding of how vaccination can be optimized across age groups, our findings enable policymakers to develop timely responses that mitigate both the cases and medical burdens of seasonal outbreaks. While our model is calibrated to the Korean context, the modeling framework itself can be applied to other settings by replacing demographic structures and healthcare capacities. Additionally, we acknowledge that alternative assumptions, such as rapid waning immunity or a mismatch between the vaccine strain and circulating variants, could alter the optimal strategy, likely necessitating vaccination schedules that are more tightly synchronized with the epidemic peak. These strategies ensure that high-priority populations, such as the elderly, are effectively protected even under limited resource constraints. Furthermore, identifying the optimal timing and distribution of vaccines is crucial for developing future delivery plans, particularly in the face of rapidly evolving influenza strains.

### Policy recommendations

Our results provide a basis for refining effective vaccination strategies, particularly regarding:

- Targeted and timely administration of vaccines to high-risk populations before the peak of the epidemic.
- Optimization of vaccination schedules using existing supplies, while accounting for healthcare system constraints such as hospital bed capacity.
- Prioritization of vaccination timing for the elderly, who exhibit the highest rates of vaccination coverage, hospitalization, and fatality, to maximize the protective impact of the program

These recommendations are directly supported by our simulation results, which demonstrate that synchronized timing with healthcare capacity can significantly reduce the peak burden of influenza.

### Limitations

First, as this study does not account for the waning of immunity following natural recovery, long-term predictions regarding vaccination strategies are limited. Accurate modeling of post recovery immunity would require simultaneous consideration of antibody dynamics and viral genotypes, which evolve annually [38–41]. Second, this study applied the POLYMOD contact matrix commonly used in age-structured influenza modeling [18,19,25]. However, this matrix may not fully capture the actual contact structure in Korea [36,37]. This limitation reflects current data constraints and highlights the potential for future extensions of the model as representative non-COVID-19 contact data for the Korean population become available. Third, our model does not include a separate compartment for pregnant women. Although the Korean National Immunization Program (KNIP) identifies pregnant women as a priority group, we were unable to quantify the specific impact of their vaccination on the overall disease burden. Fourth, the lack of vaccination data stratified by five-year age intervals necessitated the use of four broad age groups. This reduced age resolution may not fully capture the variability in vaccine uptake and effectiveness across different ages, potentially affecting the precision of our impact estimates. Finally, future studies should extend upon this modeling framework by explicitly accounting for waning immunity and asymptomatic infection, while allowing for finer age stratification and strain-dependent transmission dynamics.

## Supporting information

S1 File Supplementary file. (PDF)
