# Supplementary material for: Vaccination scenario-based study on seasonal influenza in Republic of Korea
Source: PLoS One. 2026 Apr 20;21(4):e0322686. doi: 10.1371/journal.pone.0322686 (PMC13095101; doi:10.1371/journal.pone.0322686)
Supplement: S1 File — (PDF) [file pone.0322686.s001.pdf]

Supporting information for "Vaccination scenario-based study on seasonal influenza in Republic of Korea"

Jongmin Lee[1,‡], Vijay Pal Bajiya[1,‡], Eunok Jung[1,*],

[1] Department of Mathematics, Konkuk University, Seoul, Korea

‡ These authors contributed equally to this work.
* junge@konkuk.ac.kr

Supplementary 1 Model output for estimated parameters

Fig S1 presents the cumulative confirmed cases and deaths for specific age groups (G1–G4) and the overall population. Regarding cumulative cases, G1 exhibits the highest proportion of infected cases (blue bars) at approximately 25%, significantly exceeding the overall average of roughly 6%. However, in terms of absolute numbers (orange bars), G2 accounts for the largest number of total infections (around 1.5 million), likely reflecting its larger population size despite a lower infection percentage compared to G1.

For cumulative deaths, G4 exhibits the highest severity by a wide margin. Despite having the lowest infection rate in panel (a), G4 shows the highest Case Fatality Rate (CFR) at nearly 0.6% (blue bar) and represents most cumulative deaths (orange bar), exceeding 600. In contrast, G1, which had the highest infection rate, shows a negligible fatality rate and very few deaths.

The seasonal influenza outbreak remained slow until late October 2023, followed by a rapid surge (exponential growth) through December 2023 and January 2024. The curves plateau by February 2024, marking the end of the wave. The sharp rise in the G1 curve confirms that this group drove the rapid spread of infections during the peak of the outbreak.

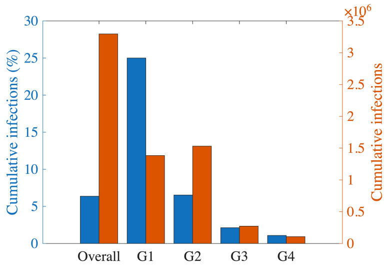

(a) Cumulative cases and percentage of cumulative cases in each group and overall also.

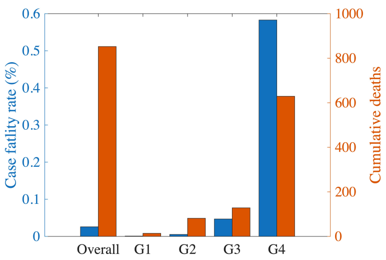

(b) Cumulative deaths and case fatality rate for each group and overall.

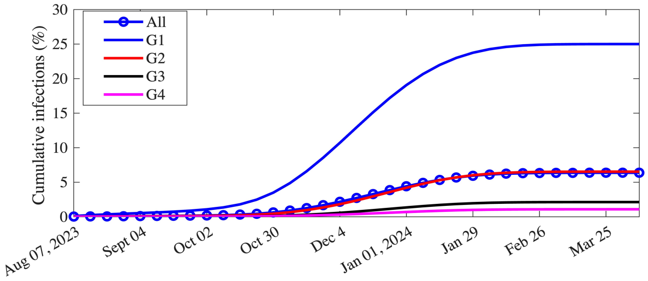

(c) Percentage of cumulative cases in each group and overall also as time varies.

Fig S1. Model output for estimated parameters ($\beta_j,\ t_0,\ s,$ and α_0).

The bootstrapping analysis [1, 2], visualized through the distribution of estimated parameters across 1,000 resamples, reveals a distinct age-dependent transmission. The transmission rate for G1 group (β_1) was estimated at a mean of 3.7284 (95% CI: 3.6169–3.8334), which is nearly seven times higher than that of G2 (β_2, mean: 0.5412) and over 180 times higher than G4 (β_4, mean: 0.0203). This substantial disparity identifies children as the primary drivers of influenza transmission within the model, whereas G4 have small contribution to the spread of infection despite being a high-risk group for severe outcomes.

Regarding seasonal forcing, the model estimated a strong seasonal amplitude (α_0) of approximately 0.58 and a sharpness parameter (s) near 1.0, indicating significant fluctuation in transmission rates throughout the year. The phase of seasonality (t_0) was precisely estimated at a mean of 15.4 weeks (95% CI: 15.31–15.47), pinpointing the timing of the epidemic peak with high accuracy. Crucially, the narrow confidence intervals observed across all parameters demonstrate that the model parameters are statistically well-identified which is evidenced by the tight clustering in the histograms. This low variance indicates that the model is robust to data fluctuations, providing high confidence in the reliability of the simulations and the derived policy recommendations.

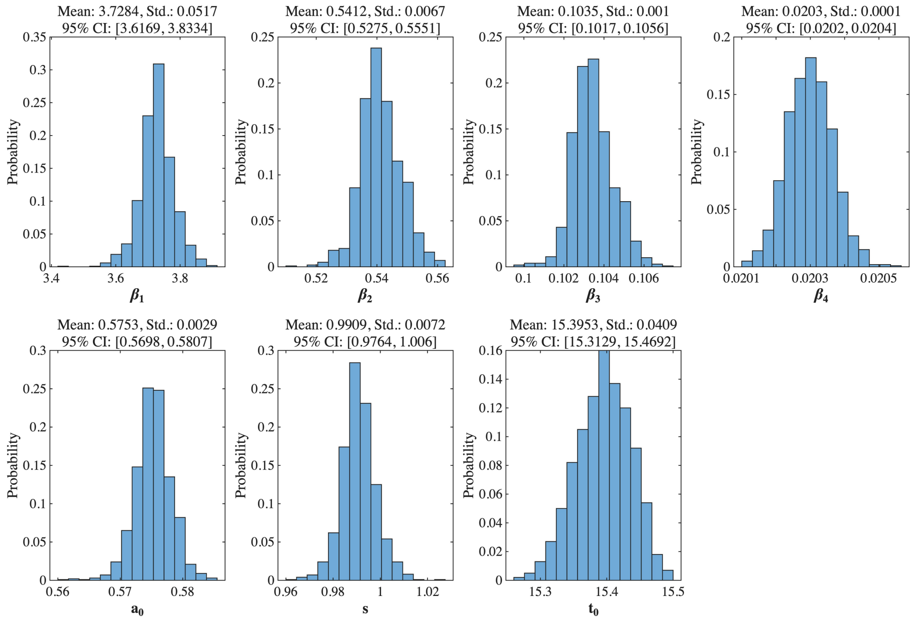

Fig S2. Bootstrap results for estimated parameters (β_j, t_0, s, and α_0).

Supplementary 2 Impact of vaccination timing on cumulative deaths

Fig S3 presents the impact of vaccination timing on cumulative deaths across different population groups. The analysis is organized into three distinct scenarios, represented by three columns: shifting the schedule for all groups simultaneously (left), shifting only Group 1 (middle), and shifting only Group 4 (right). The top row provides a schematic of these strategies, using a Shifting box to indicate which group's vaccination schedule is moved relative to the standard start dates (e.g., Oct 9th).

The left column demonstrates that advancing the timeline for the entire population has a profound impact on mortality outcomes. The bar charts show that shifting the schedule two weeks earlier (-2w, blue bars) significantly reduces overall cumulative deaths compared to the baseline (0, yellow bars). Conversely, a delay of just two weeks (+2w, green bars) results in a sharp surge in mortality. In the bottom panel, line graphs quantify these deviations; an early start reduces deaths by nearly 50%, while a two-week delay drives an increase of over 75% compared to the baseline.

In contrast, the middle column reveals that altering the timing for G1 alone yields a negligible effect on overall mortality. The cumulative death bars remain nearly uniform in height regardless of whether the vaccination timing for G1 is advanced or delayed. Similarly, the percentage change graph in the bottom row is near flat, fluctuating close to zero. This suggests that while G1 may be a high-transmission group, the timing of their intervention has little direct influence

on the total death toll, emphasizing the need for broader or more targeted strategies for high-risk groups.

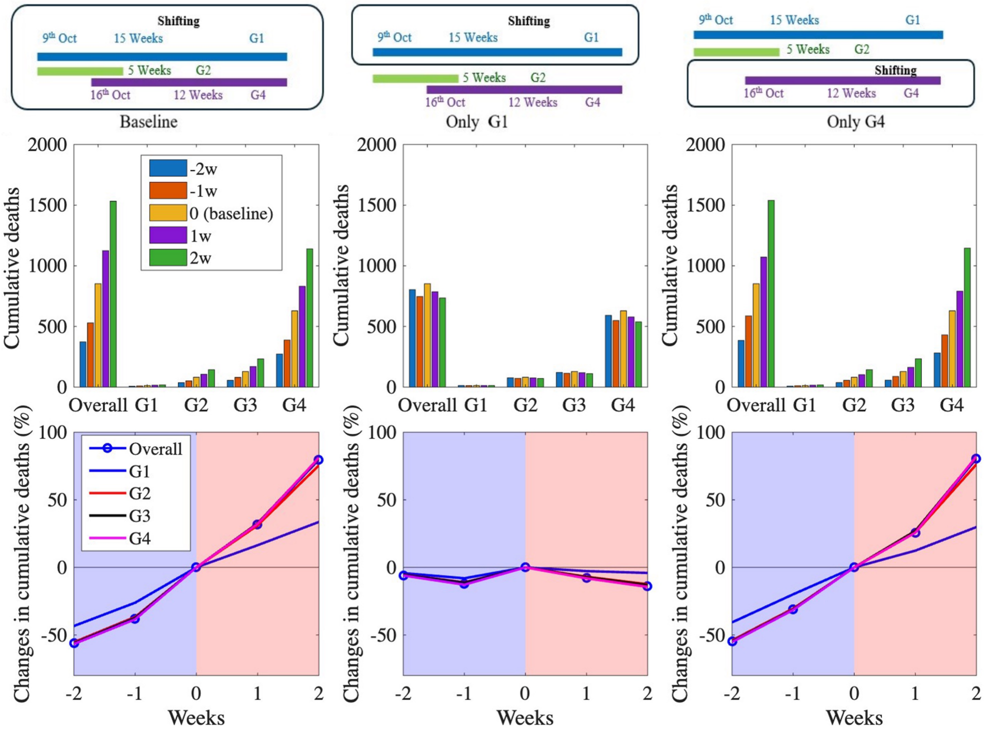

Fig S3. Impact of vaccination timing on cumulative deaths (top) and changes in cumulative deaths (%) (bottom).

The right column identifies G4 as the critical driver of cumulative deaths. Shifting the vaccination schedule for G4 alone produces a pattern nearly identical to that of shifting the entire population, where early intervention saves substantial lives and delays cause a sharp rise in mortality. This confirms that the timing of protection for the vulnerable G4 population is the decisive factor in reducing fatalities, whereas the scheduling for groups like G1 is far less consequential for reducing deaths.

Table S6 presents a summary of the results shown in Fig S3, including absolute values and the corresponding reductions (or increases) compared to the baseline scenario.

Table S1. Impact of varying Simulation results of vaccination timing on death cases

Scenario	Age group	2 weeks faster	1 week faster	Baseline	1 week later	2 weeks later
All group	Overall	373 (-56.3%)	529 (-37.9%)	852	1124 (31.9%)	1532 (79.8%)
	G1	8 (-43.4%)	10 (-26.1%)	14	16 (16.4%)	18 (33.6%)
	G2	36 (-55.3%)	52 (-36.7%)	81	107 (31.1%)	143 (75.5%)
	G3	56 (-56.2%)	80 (-37.5%)	128	170 (32.6%)	232 (80.8%)
	G4	272 (-56.7%)	387 (-38.4%)	629	831 (32.2%)	1139 (81.1%)
Only G1	Overall	803 (-5.8%)	747 (-12.4%)	852	786 (-7.8%)	734 (-13.9%)
	G1	13 (-4.3%)	13 (-8.1%)	14	13 (-2.8%)	13 (-4.1%)
	G2	77 (-5.2%)	72 (-11.0%)	81	76 (-7.0%)	71 (-12.5%)
	G3	121 (-5.4%)	114 (-11.4%)	128	119 (-7.2%)	112 (-12.9%)
	G4	591 (-6.0%)	548 (-12.8%)	629	578 (-8.1%)	538 (-14.4%)
Only G4	Overall	385 (-54.8%)	587 (-31.2%)	852	1072 (25.7%)	1540 (80.6%)
	G1	8 (-40.7%)	11 (-19.9%)	14	15 (12.4%)	18 (29.9%)
	G2	37 (-54.0%)	57 (-30.4%)	81	102 (25.8%)	143 (76.2%)
	G3	58 (-54.9%)	88 (-31.2%)	128	163 (27.0%)	233 (81.6%)
	G4	282 (-55.2%)	431 (-31.5%)	629	791 (25.7%)	1145 (82.1%)

Supplementary 3 Vaccination timing scenarios on cumulative death

Fig S4 illustrates the effects of different vaccination timing scenarios on mortality across distinct subpopulations and the total population. The bar chart on the left presents cumulative deaths under three scenarios (S0, S1, and S2), showing that S0 consistently yields the highest mortality, while S2 produces the greatest reduction in cumulative deaths for both the overall population and subgroups G1–G4. The panel on the right quantifies these differences by reporting the percentage change in cumulative deaths for scenarios S1 and S2 relative to the baseline (S0). Under scenario S1, the reduction in mortality ranges from approximately 20% to 32% across the subgroups. In contrast, scenario S2 produces a substantially larger impact, with reductions in cumulative deaths exceeding 50% for nearly all categories, including the overall population and subgroups G2, G3, and G4.

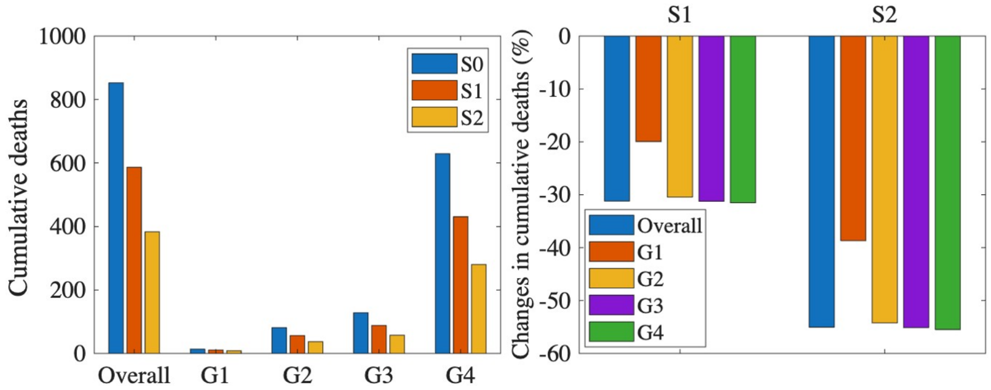

Fig S4. Cumulative deaths for three different vaccine scenarios and changes in cumulative deaths for S1 and S2 compared to S0 (baseline).

Supplementary 4 Model output for vaccination scenarios and immunity levels

Fig S5 presents the modeled weekly number of confirmed cases over a 35-week horizon under three vaccination timing scenarios (S0, S1, S2) and three levels of immunity (L1, L2, L3). In the baseline scenario (S0), which assumes no modification to the vaccination schedule for the G4 group, the epidemic peak attains its maximum magnitude. Within this baseline, the S0–L1 combination (blue curve) serves as the default, most closely reproducing the empirical observations (indicated by red markers). Advancing the vaccination schedule for the G4 group by one week (S1) and two weeks (S2) yields a monotonic and noticeable decrease in peak incidence. Furthermore, across all vaccination scenarios, higher levels of immunity are systematically associated with lower number of confirmed cases. This effect is most pronounced in the L3 assumption, which incorporates both reported and unreported infections over the preceding two years, thereby producing substantially lower projected case counts compared to the more conservative L1 and L2 estimates.

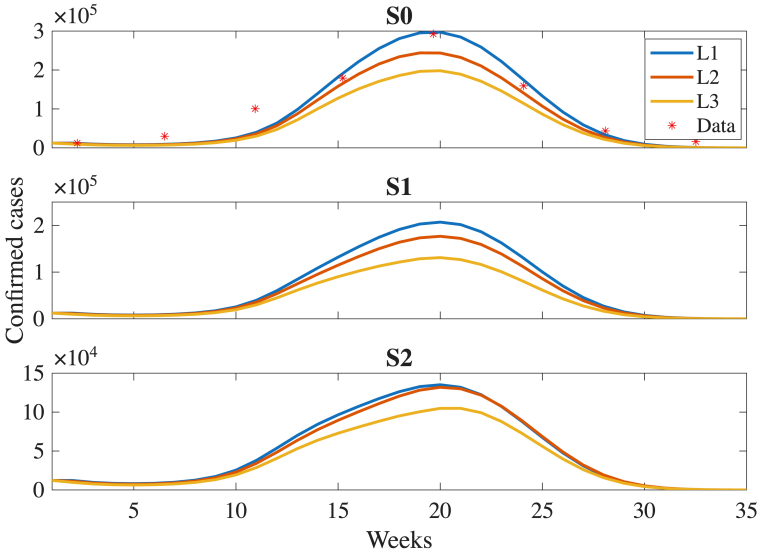

Fig S5. Cumulative cases for three different vaccination scenarios and immunity levels as time vary.

Supplementary 5 Reduction in the peak size of cumulative cases

Fig S6 presents the percentage reduction in the peak number of infections across various vaccination and immunity scenarios, using the S0–L1 combination as the baseline for comparison. Under the default vaccination schedule (no change in vaccination timing), increasing pre-existing immunity from L1 to L2 and L3 yields peak reductions of 17.91% and 33.31%, respectively. Advancing the vaccination schedule for the G4 group produces substantially larger effects; for example, the S1 scenario (vaccination initiated one week earlier) combined with L3 immunity results in a 55.92% reduction in the peak, whereas the S2 scenario (two weeks earlier) combined with L3 immunity attains the maximum observed peak reduction of 64.72%. Even under the lowest assumed immunity level (L1), shifting from the baseline to the S2 vaccination schedule alone produces a marked 54.54% decrease in the infection peak.

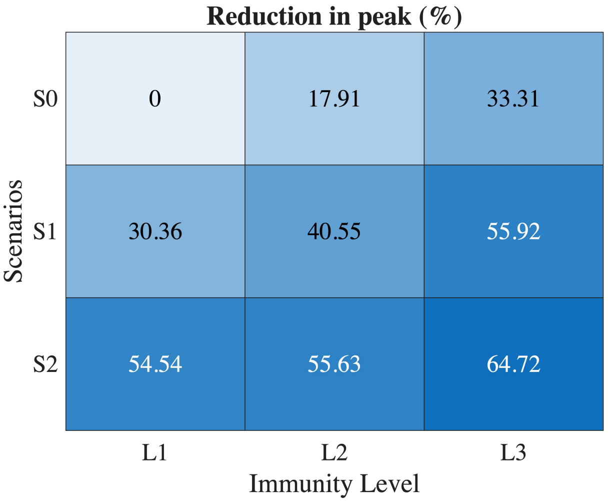

Fig S6. Percentage reduction in the peak size of cumulative cases in different vaccination and immunity levels. Here, S0-L1 is considered as the baseline case.

Supplementary 6 Duration (in weeks) for that the hospitalized exceeds the particular threshold

Table S2 summarizes the projected number of weeks during which hospitalizations exceed predefined critical thresholds, categorized by vaccination schedule and baseline population immunity based on Fig 9. For a hospitalization threshold of 4000, the duration above this level ranges from a maximum of 13 weeks under the baseline S0-L1 and S0-L2 scenarios to a minimum of 6 weeks under the S2-L3 scenario, which combines a two-week advancement in vaccine rollout with the highest level of pre-existing immunity. At the higher threshold of 5000 hospitalizations, the beneficial effect of accelerated vaccination is even more marked; for example, the duration above this level decreases from 13 weeks in the baseline S0-L1 scenario to 0 weeks in the S2-L3 scenario, indicating that hospitalizations never reach this critical level under the most favorable conditions. Collectively, these results indicate that both advancing the timing of vaccination and incorporating higher levels of prior immunity substantially diminish the period during which the healthcare system experiences extreme stress.

		Vaccination Scenarios		
Immunity		**S0**	**S1**	**S2**
	**L1**	13	12	10
	**L2**	13	11	9
	**L3**	12	9	6

(a) When hospitalized threshold = 4000

		Vaccination Scenarios		
Immunity		**S0**	**S1**	**S2**
	**L1**	13	11	7
	**L2**	11	9	6
	**L3**	10	5	0

(b) When hospitalized threshold = 5000

Table S2. Duration (in weeks) for that the hospitalized exceeds the particular threshold

Supplementary 7   Contact matrix

The estimated contact matrix for Korea, as presented in the work of Prem et al. [3], was derived using population-based contact diaries from the European POLYMOD survey. This survey was utilized to generate projections for various countries, including Korea. These contact matrices contain contact rates across 16 age categories, which are organized in five-year increments, covering the age range from 0 to 80 years. However, for the purposes of our model, we focus on only four broader age groups. To achieve this, we aggregated the original data into four distinct age groups: 0-14, 15-49, 50-64, and 65-80. This aggregation was done by calculating population-weighted sums across the individual age classes within each of these broader groups. We used the following population-weighted sums:

$$\bar{C}_{j,k} = \sum_{j' \in j} \left(\frac{N_{j'}}{N_j} \sum_{k' \in k} C_{j',k'} \right) \tag{1}$$

where $\{j', k'\}$ are the subscripts for the five-year age intervals, $\{j, k\}$ are the subscripts for the larger age intervals as we considered, $C_{j',k'}$ represents the average number of daily contacts a person in age group j' makes with that of group k' and $N_{j'}$ is the total population of age group j'.

The total number of j to k contacts must be equal to those of k to j: $N_j C_{j,k} = N_k C_{k,j}$. Due to numerical challenges encountered during estimation, such as bin discretization and rounding errors, small discrepancies can arise in the results. To address this, we take precautionary measures to ensure the condition is consistently met by implementing specific conditions. This helps minimize the impact of such issues and ensures the desired outcome is achieved. We ensure this condition holds by imposing

$$C_{j,k} = \frac{0.5 \left(N_j \bar{C}_{j,k} + N_k \bar{C}_{k,j} \right)}{N_j} \tag{2}$$

In this equation, the numerator represents the average of the two measures of total contacts between groups j and k, while the denominator adjusts the result to a per-capita basis for group j. From this process, we have the contact matrix as depicted in Fig S7.

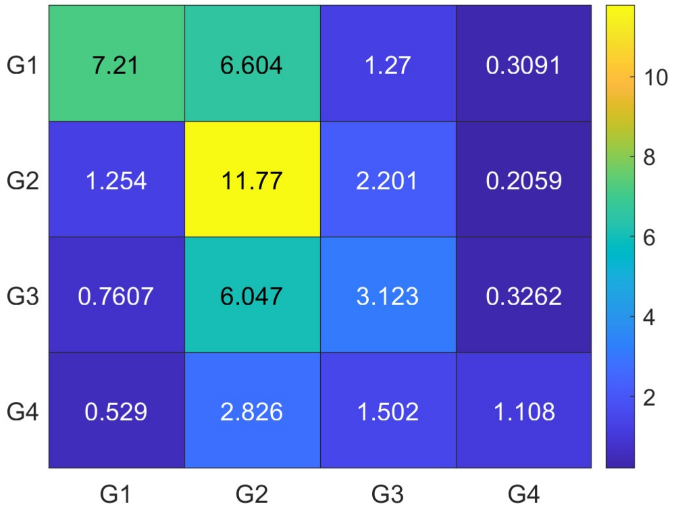

Fig S7. Contact for four age groups.

Supplementary 8   Initial Conditions

To estimate the initial size of the various model compartments, we utilized the monthly confirmed influenza case data from the HIRA [4,5] and weekly data from the FluNet surveillance system of the WHO [6]. Upon comparing both

datasets, we observed that they followed similar patterns. This allowed us to distribute the monthly HIRA data into weekly intervals to derive the confirmed case data for the first week of August 2023.

From this confirmed case data for the first week of August 2023, we estimated the number of exposed individuals at the initial time, $t = 0$ (i.e., the first week of August). The calculation of the exposed individuals was based on the confirmed cases at $t = 0$, using the relationship:

$$\text{Confirmed Cases } (at \;\; t = 0) = q\kappa E_j(0)$$

where $j = 1, 2, 3, 4$ corresponds to the different age groups. To further refine the model, we assumed that the confirmed cases observed in the first week of August 2023 were indicative of a stable state. Hence, we assumed that the rate of change in the infected compartment, $\frac{dI_j}{dt} = q\kappa E_j - (1 - q_{hj})\alpha I_j - q_{hj}\rho I_j$ and $\frac{dA_j}{dt} = (1 - q)\kappa E_j - \eta A_j$ were zero at $t = 0$. This assumption allowed us to calculate the initial population sizes of the infected (I_j) and asymptomatic $(A_j\;)$ individuals for each age group at $t = 0$ as equation (3).

$$\begin{aligned}
q\kappa E_1(0) &= 26180/31 = 845,\; E_1(0) = 2397\\
q\kappa E_2(0) &= 20364/31 = 657,\; E_2(0) = 1863\\
q\kappa E_3(0) &= 4797/31 = 155,\; E_3(0) = 440\\
q\kappa E_4(0) &= 2986/31 = 96,\; E_4(0) = 272\\
q\kappa E_1(0) &= (\alpha - \alpha q_{h1} + \rho q_{h1})I_1(0),\; I_1(0) = 4807\\
q\kappa E_2(0) &= (\alpha - \alpha q_{h2} + \rho q_{h2})I_2(0),\; I_2(0) = 3754\\
q\kappa E_3(0) &= (\alpha - \alpha q_{h3} + \rho q_{h3})I_3(0),\; I_3(0) = 854\\
q\kappa E_4(0) &= (\alpha - \alpha q_{h4} + \rho q_{h4})I_4(0),\; I_4(0) = 480\\
(1 - q)\kappa E_1(0) &= \eta A_1(0),\; A_1(0) = 2498\\
(1 - q)\kappa E_2(0) &= \eta A_2(0),\; A_2(0) = 1941\\
(1 - q)\kappa E_3(0) &= \eta A_3(0),\; A_3(0) = 458\\
(1 - q)\kappa E_4(0) &= \eta A_4(0),\; A_4(0) = 283
\end{aligned} \tag{3}$$

where all time unit of the parameters are converted to day.

For the calculation of the recovered population, we took into account the assumption that individuals who had been confirmed as cases in the previous year were likely to have developed immunity to influenza in the current year. Therefore, the confirmed cases from the previous year were considered as the recovered population for the current model.

Regarding the vaccination-related compartments, we assumed that there were no vaccinations administered during the month of August 2023, so these compartments were considered to have a population size of zero. Finally, the remaining individuals in each age group, who had not been classified as exposed, infected, or recovered, were considered as susceptible individuals within their respective age groups.

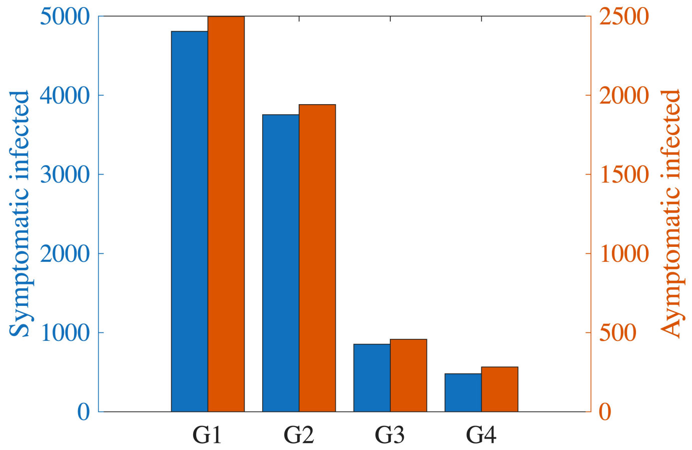

Fig S8. Initial infected population for each age group.

Supplementary 9 Sensitivity analysis

We conducted a partial rank coefficient correlation (PRCC) analysis [7–9] to investigate the sensitivity of model parameters with respect to three major outputs: cumulative confirmed cases, hospitalized cases, and cumulative deaths. Vector-valued parameters q_h, σ, and d were analyzed using a scale parameter, defined as a constant that scales the baseline values within a range of 0-to 2-fold with the parameters q_h^{mag}, σ^{mag}, and d^{mag}, respectively. Table S3 shows the parameter range used for the PRCC sensitivity analysis along with the values used in the default simulation.

Table S3. Parameter range for sensitivity analysis

Symbol	Baseline value	Range	Symbol	Baseline value	Range
β_1	3.7062	[0,10]	β_2	0.5346	[0,1]
β_3	0.1023	[0,1]	β_4	0.0203	[0,1]
a_0	0.5743	[0,1]	s	0.9866	[0,2]
t_0	15.3738	[10,30]	δ	0.5	[0,1]
κ	3.6842	[0,7]	q	0.67	[0,1]
η	1.1667	[0,7]	α	1.1667	[0,7]
ω	0.7	[0,7]	ρ	7	[0,14]
γ	0.0389	[0,0.1167]	q_h^{mag}	1	[0,2]
σ^{mag}	1	[0,2]	d^{mag}	1	[0,2]
dummy	1	[0,2]			

The sensitivity analysis, evaluated at the 2nd, 11th, and 35th weeks, reveals that parameter influence is most pronounced during the early phase of the epidemic (2nd week) and generally diminishes over time. The parameter κ exhibits a temporal shift from a positive correlation in the early phase to a negative correlation by the end of the epidemic. For cumulative confirmed cases, the proportion of symptomatic individuals (q) and the transmission rate among the elderly (β_4) were the strongest positive drivers, indicating that higher symptomaticity and increased transmission within the high-risk elderly group substantially amplify the total infection burden. Conversely, the recovery rate of symptomatic individuals (α) and the recovery rate of asymptomatic individuals (η) showed strong negative correlations, suggesting that faster recovery effectively curtails viral spread.

Regarding severe outcomes, the dominant drivers shifted toward clinical parameters. For hospitalized cases, the age-specific proportion of symptomatic individuals requiring hospitalization (q_h) and the rate of transition to hospitalization (ρ) emerged as the primary positive factors, alongside β_4. Cumulative deaths were most sensitive to the mortality rate (d), followed closely by q_h and ρ. Notably, the recovery rate of hospitalized individuals (σ) exhibited a strong negative association with both hospitalizations and deaths, underscoring the critical role of hospital treatment efficacy in reducing severe outcomes. By the 35th week (yellow bars), the influence of transmission-related parameters wanes, whereas parameters governing fatality and recovery become increasingly important.

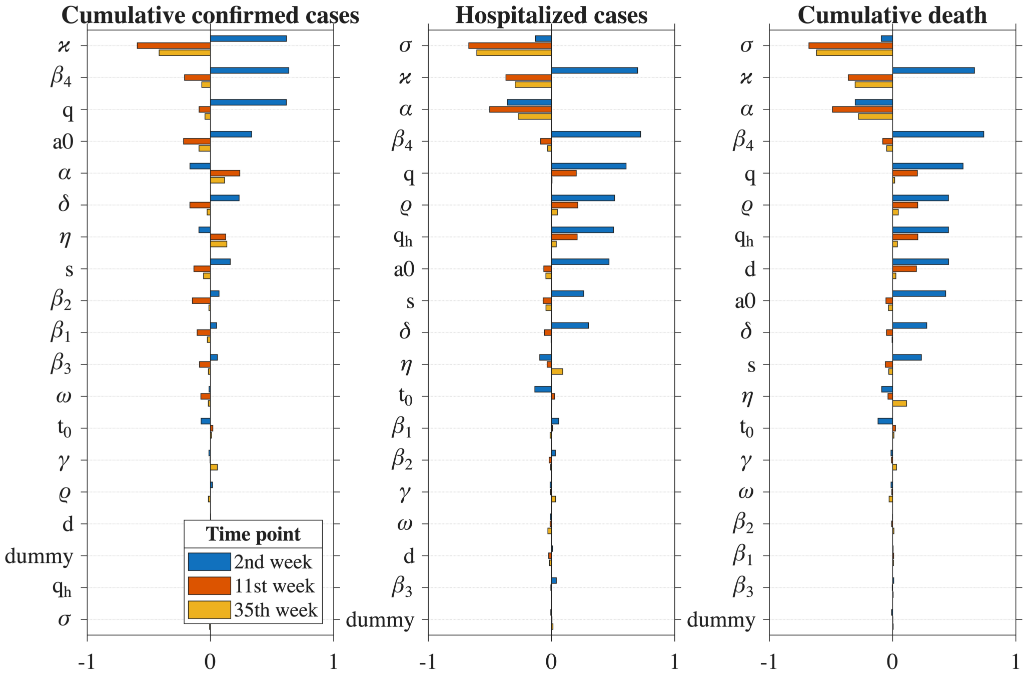

Fig S9. PRCC results for three major outputs.

References

1. Kim RH, Day SC, Small DS, Snider CK, Rareshide CA, Patel MS. Variations in influenza vaccination by clinic appointment time and an active choice intervention in the electronic health record to increase influenza vaccination. JAMA network open. 2018;1(5):e181770–e181770.

2. Cooper BS, Pitman RJ, Edmunds WJ, Gay NJ. Delaying the international spread of pandemic influenza. PLoS medicine. 2006;3(6):e212.

3. Prem K, Cook AR, Jit M. Projecting social contact matrices in 152 countries using contact surveys and demographic data. PLOS Computational Biology. 2017;13(9):1–21. doi:10.1371/journal.pcbi.1005697.

4. HIRA. National Health Concern Disease Statistics - aged population sample - Influenza;. Available from: `https://opendata.hira.or.kr/op/opc/olapMfrnIntrsIlnsInfoTab2.do`.

5. Kim JA, Yoon S, Kim LY, Kim DS. Towards Actualizing the Value Potential of Korea Health Insurance Review and Assessment (HIRA) Data as a Resource for Health Research: Strengths, Limitations, Applications, and Strategies for Optimal Use of HIRA Data. J Korean Med Sci. 2017;32(5):718–728. doi:10.3346/jkms.2017.32.5.718.

6. WHO. WHO Influenza (seasonal) `https://worldhealthorg.shinyapps.io/flunetchart/`; 2024.

7. Marino S, Hogue IB, Ray CJ, Kirschner DE. A methodology for performing global uncertainty and sensitivity analysis in systems biology. Journal of Theoretical Biology. 2008;254(1):178–196. doi:https://doi.org/10.1016/j.jtbi.2008.04.011.

8. Samsuzzoha M, Singh M, Lucy D. Uncertainty and sensitivity analysis of the basic reproduction number of a vaccinated epidemic model of influenza. Applied Mathematical Modelling. 2013;37(3):903–915.

9. Gilbert JA, Meyers LA, Galvani AP, Townsend JP. Probabilistic uncertainty analysis of epidemiological modeling to guide public health intervention policy. Epidemics. 2014;6:37–45.
